# Supplementary material for: Rational design of 19F NMR labelling sites to probe protein structure and interactions
Source: Nat Commun. 2025 May 8;16:4300. doi: 10.1038/s41467-025-59105-6 (PMC12062419; doi:10.1038/s41467-025-59105-6)
Supplement: Supplementary file 1 — Supplementary Information [file 41467_2025_59105_MOESM1_ESM.pdf]

Rational design of ^{19}F NMR labelling sites to probe protein structure and interactions

Supplementary Information

Supplementary Figures

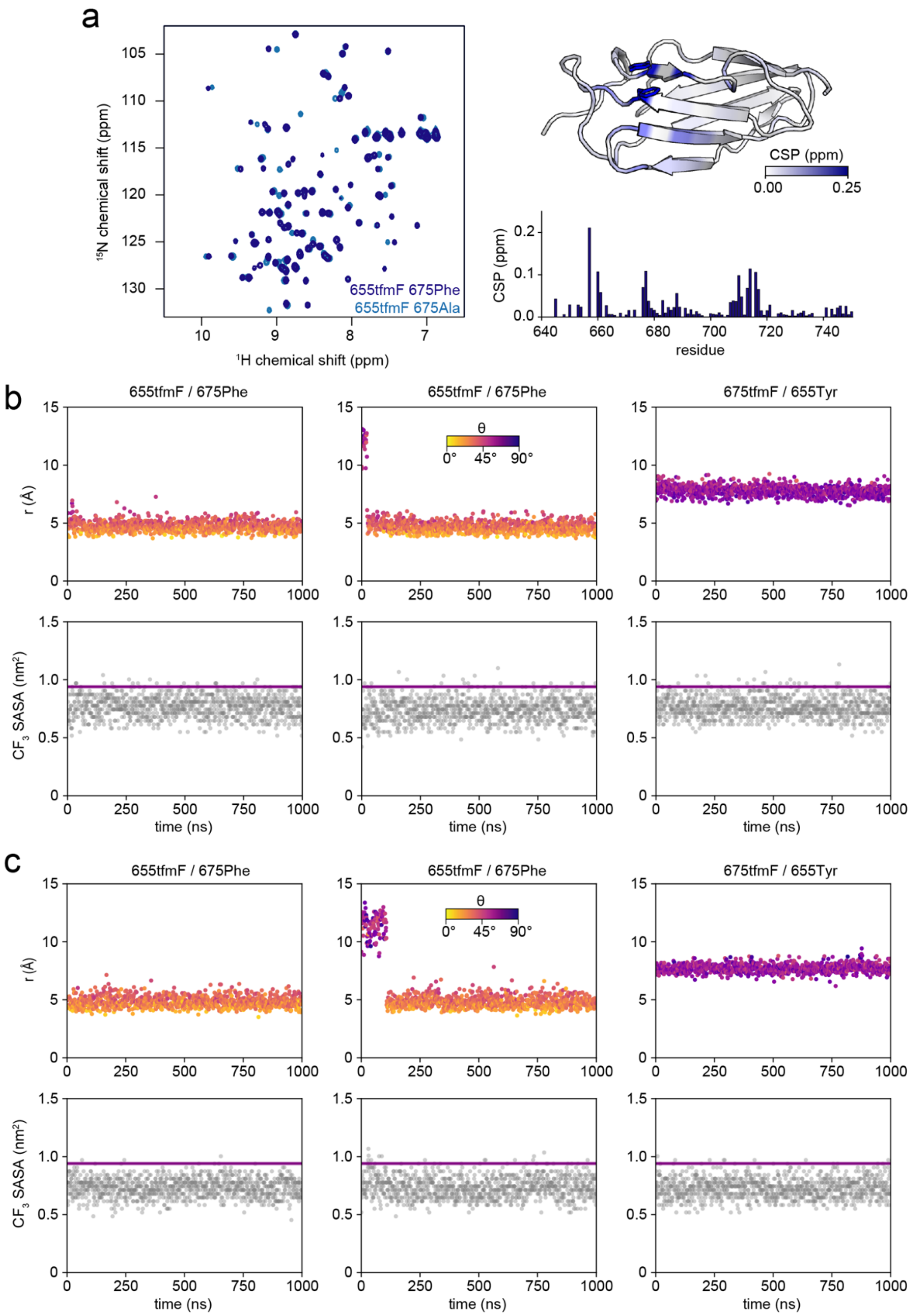

Supplementary Figure 1. Experimental and computational characterisation of tfmF-labelled FLN5. **a.** 2D ^{1}H,^{15}N SOFAST-HMQC spectra of FLN5 655tfmF with and without the F675A mutation, recorded at 298 K and 500 MHz. Residue-specific chemical shift perturbations (CSP = $[(\Delta\delta_H)^2 + (0.2 * \Delta\delta_N)^2]^{1/2}$, where $\Delta\delta_H$ and $\Delta\delta_N$ are differences in the ^{1}H and ^{15}N chemical shifts respectively) are plotted and mapped onto the FLN5 structure (PDB 1QFH). **b.** Distances (r) and angles (θ) between respective CF_3 groups and nearby aromatic rings observed in all-atom MD simulations with the ff15ipq and **c** C36m

force field. The bottom panels show the solvent-accessible surface area (SASA) of the CF_3 group during the simulation and the horizontal line represents a fully solvated CF_3 group in a disordered peptide. The middle panels show simulation results of 655tfmF/675Phe initiated from a different sidechain rotamer of residue 655.

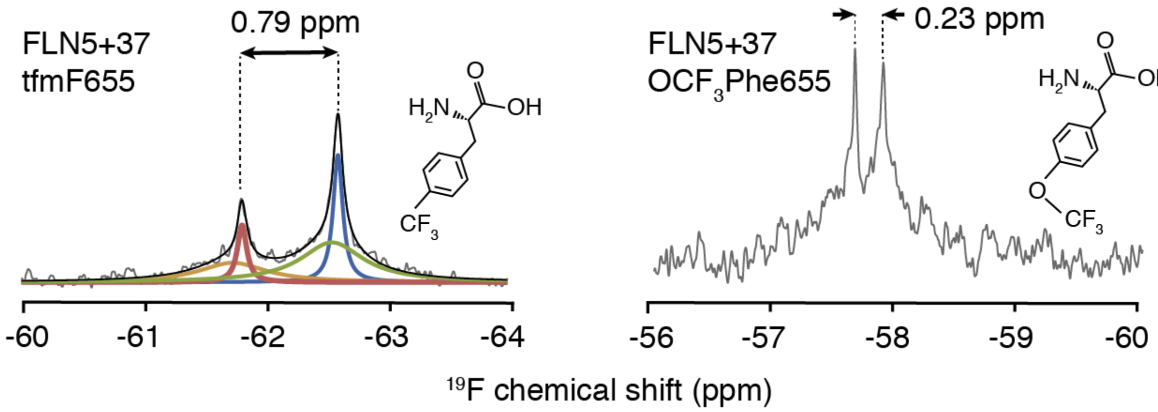

Supplementary Figure 2. ^{19}F NMR of FLN5 ribosome-nascent chain complexes labelled with tfmF and tfmOF (trifluoromethoxy-L-phenylalanine) at residue 655. Secondary chemical shifts are indicated. Spectra were recorded at 298 K and 500 MHz.

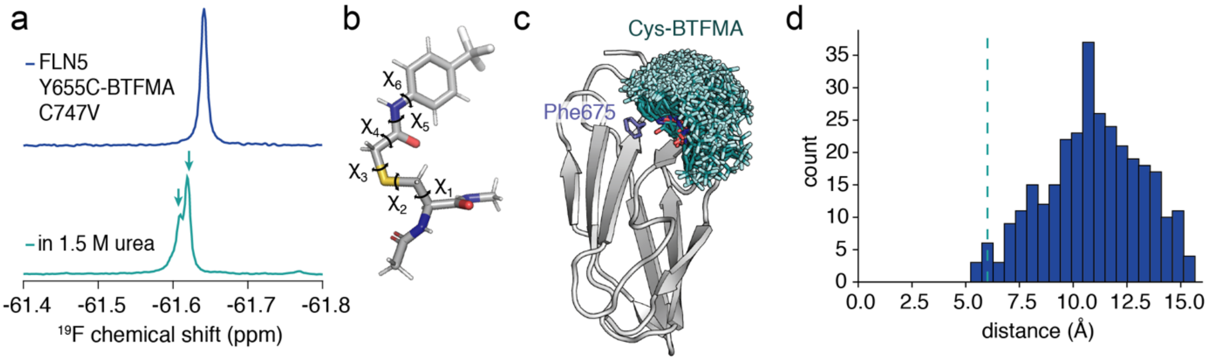

Supplementary Figure 3. BTFMA-labelled FLN5. **a.** ^{19}F NMR spectrum of FLN5 655Cys labelled with 2-bromo-N-(4-[trifluoromethyl]phenyl)acetamide (BTFMA) (with a cysteine-free background, Cys747Val) in Tico buffer (top) and buffer with 1.5 M urea (bottom). The arrows highlight two separate peaks corresponding to the unfolded and folded conformation. Spectra were recorded at 298 K and 500 MHz. **b.** Structure of a cysteine conjugated with BTFMA highlighting its rotatable sidechain dihedral angles. **c.** Cysteine-BTFMA rotamers aligned to residue 655 of the FLN5 crystal structure (PDB 1QFH) and **d.** a histogram of the CF_3-Phe675 ring distance (between the centres of mass). Dashed line indicates 6 Å, below which ring current effects are observable.

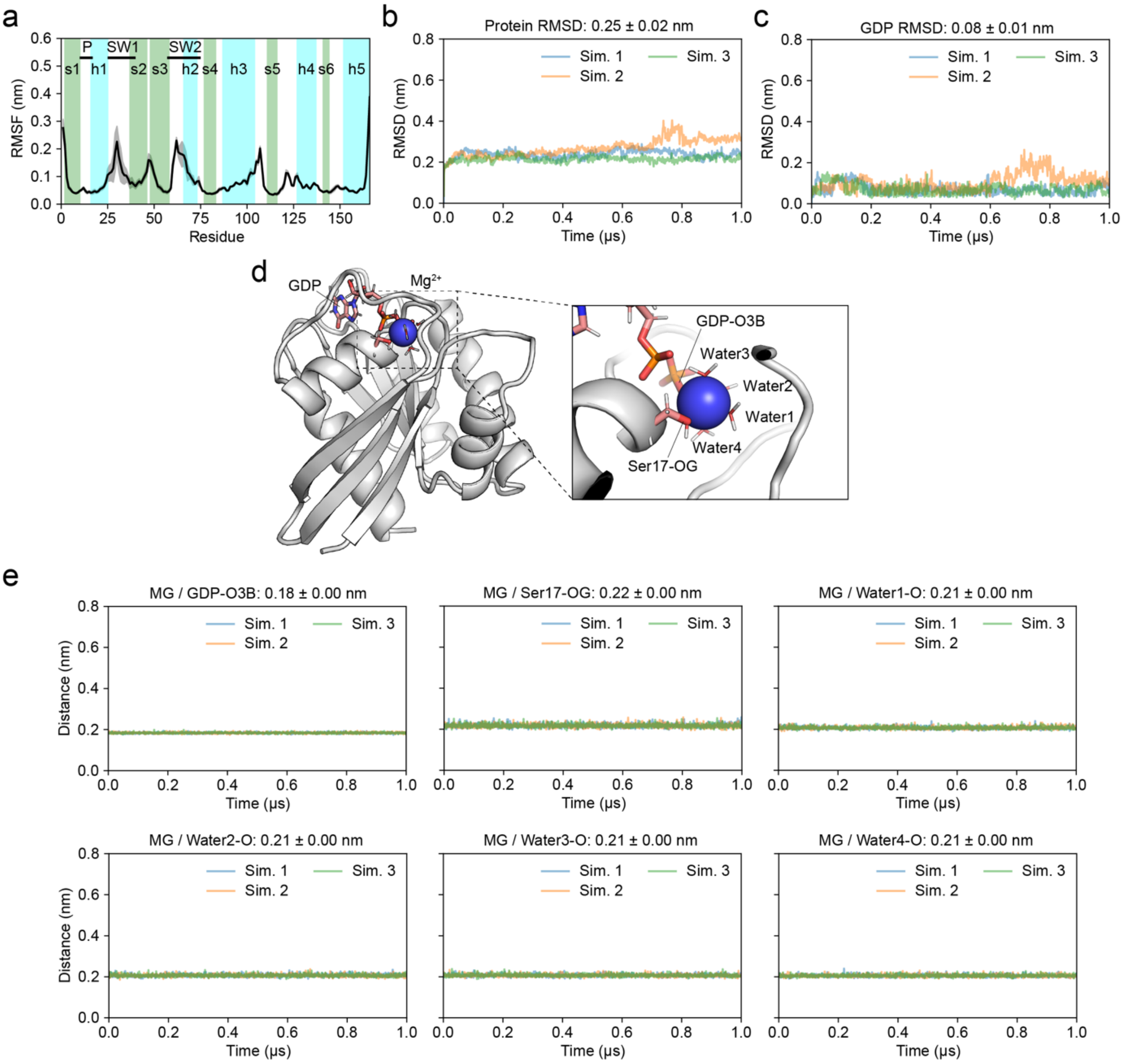

Supplementary Figure 4. All-atom MD simulations of the HRAS-GDP complex with implicitly polarised charges (IPolQ) for GDP. **a.** Average root mean square fluctuations (RMSF) for each residue Cα atom (mean ± s.e.m. from three independent 1 μs-long simulations). **b-c.** Protein and ligand (all-atom) RMSD calculated for three independent 1 μs-long simulations (mean ± s.e.m.). **d.** Energy-minimised structure of HRAS-GDP highlighting the coordination of a structural Mg^{2+} by a GDP phosphate oxygen atom, Ser17 oxygen atom and four structural water molecules. **e.** Mg^{2+} coordination distances highlighting the stability of the structural ion and ligand in the complex (mean ± s.e.m.).

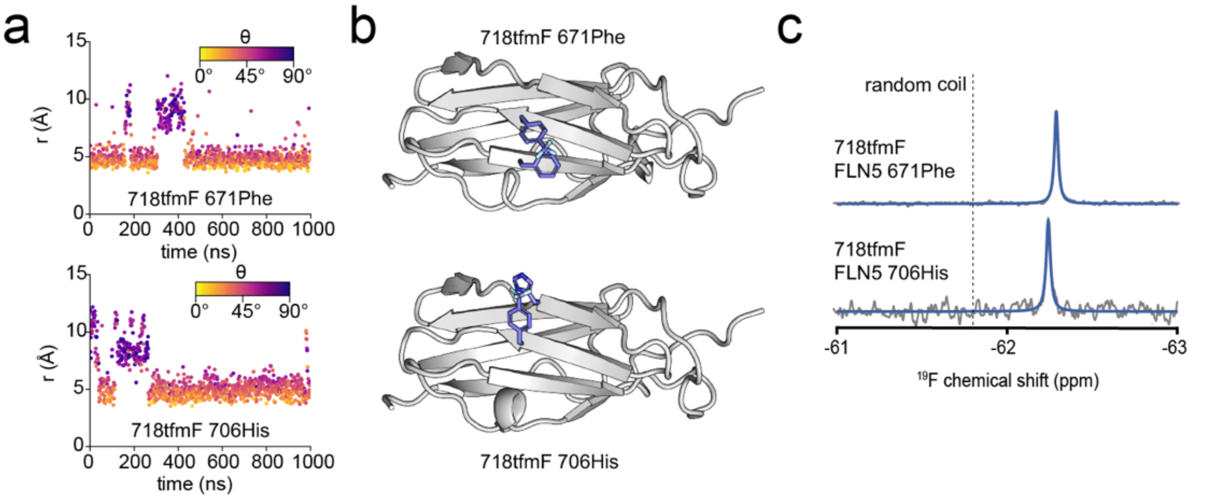

Supplementary Figure 5. Probing different structural interactions using a single fluorine labelling site on FLN5. **a.** Distance (r) and angle (θ) between the CF_3 group of 718tfmF (E-strand) and aromatic ring of 671Phe (B-strand) and 706His (D-Strand) observed in representative all-atom MD simulations. **b.** Predicted structures of FLN5 718tfmF with 671Phe and 706His. **c.** ^{19}F NMR spectra of FLN5 671tfmF with Glu671Phe and Lys706His point mutations, recorded at 298 K and 500 MHz.

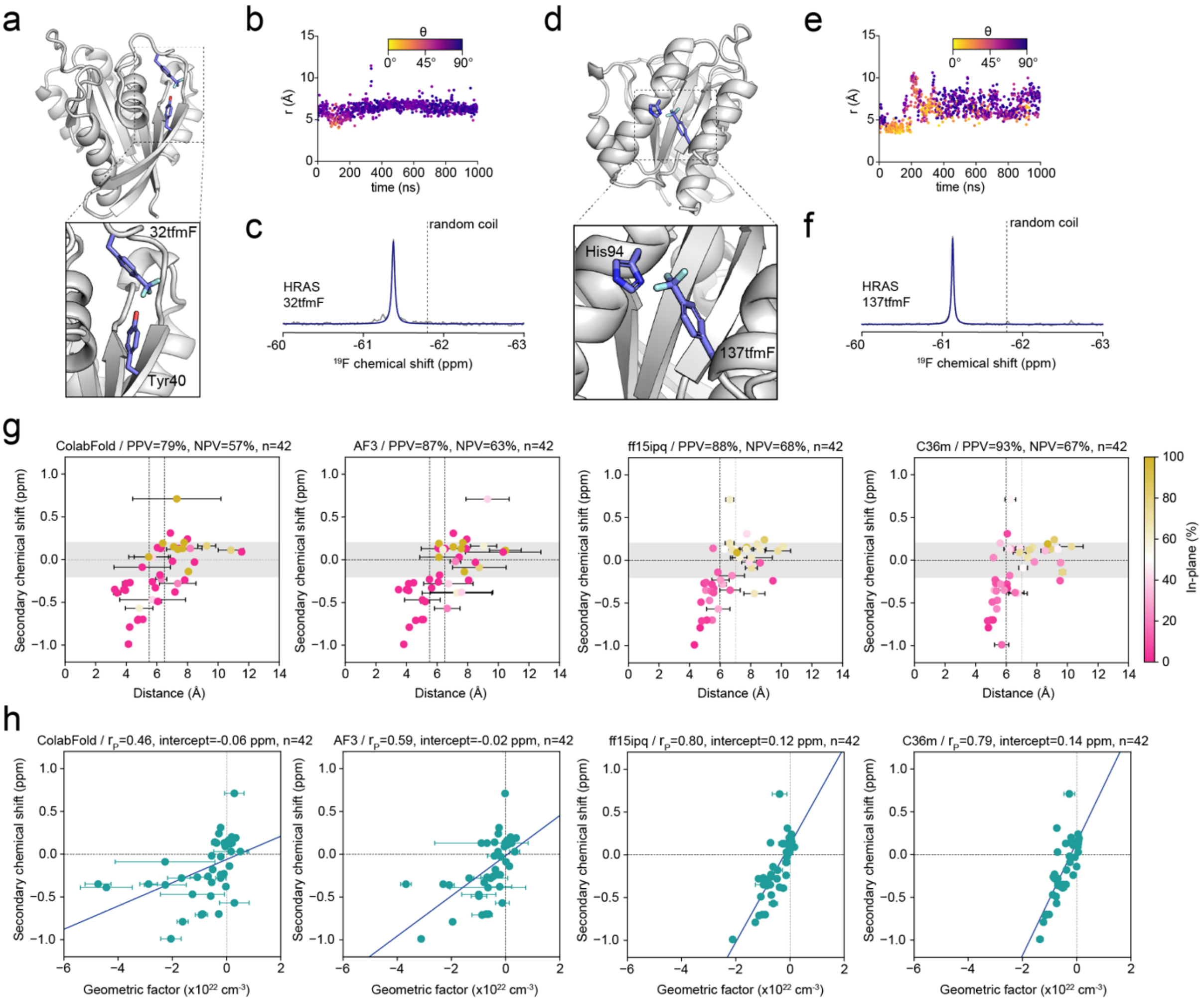

Supplementary Figure 6. Ring current design to probe protein structure using ^{19}F NMR chemical shifts. **a.** Structural model of HRAS 32tfmF, highlighting an interaction between tfmF and 40Tyr between a

loop and β-strand. **b.** Distance (r) and angle (θ) between the CF_3 group of 32tfmF and aromatic ring of 40Tyr observed in a representative all-atom MD simulation. **c.** ^{19}F NMR spectra of HRAS 32tfmF recorded at 298 K and 500 MHz. **d.** Structural model of HRAS 137tfmF highlighting an interaction between tfmF and 94His across two α-helices. **e.** Distance (r) and angle (θ) between the CF_3 group of 137tfmF and aromatic ring of 94His observed in a representative all-atom MD simulation. **f.** ^{19}F NMR spectra of HRAS 137tfmF recorded at 298 K and 500 MHz. **g-h.** Correlation analysis for a subdataset of 42 proteins that could be modelled by both MD force fields (ff15ipq and C36m). **g.** Scatter plots correlating the distances (coloured by the fraction of time (MD) or models (ColabFold/AF3) spent in the plane of the ring defined as θ > 54.6°) predicted by ColabFold, AF3, ff15ipq (MD), and C36m (MD) with secondary ^{19}F chemical shifts for all protein variants (Tables S3-S6). The error bars represent one s.d. over the five predicted models for ColabFold and AF3, and the s.e.m. obtained from three independent simulations for MD. PPV=positive predictive value; NPV=negative predictive value. Positive secondary chemical shift > 0.2 ppm in magnitude. The vertical lines represent the distance cut-off values for perpendicular and in-plane interactions (lower and higher distance, respectively). **h.** Correlations between predicted geometric factors $(1\text{-}3\cos^2\theta)/r^3$) and secondary ^{19}F chemical shifts and the corresponding Pearson correlation coefficients (r_P) and intercepts for lines of best fit.

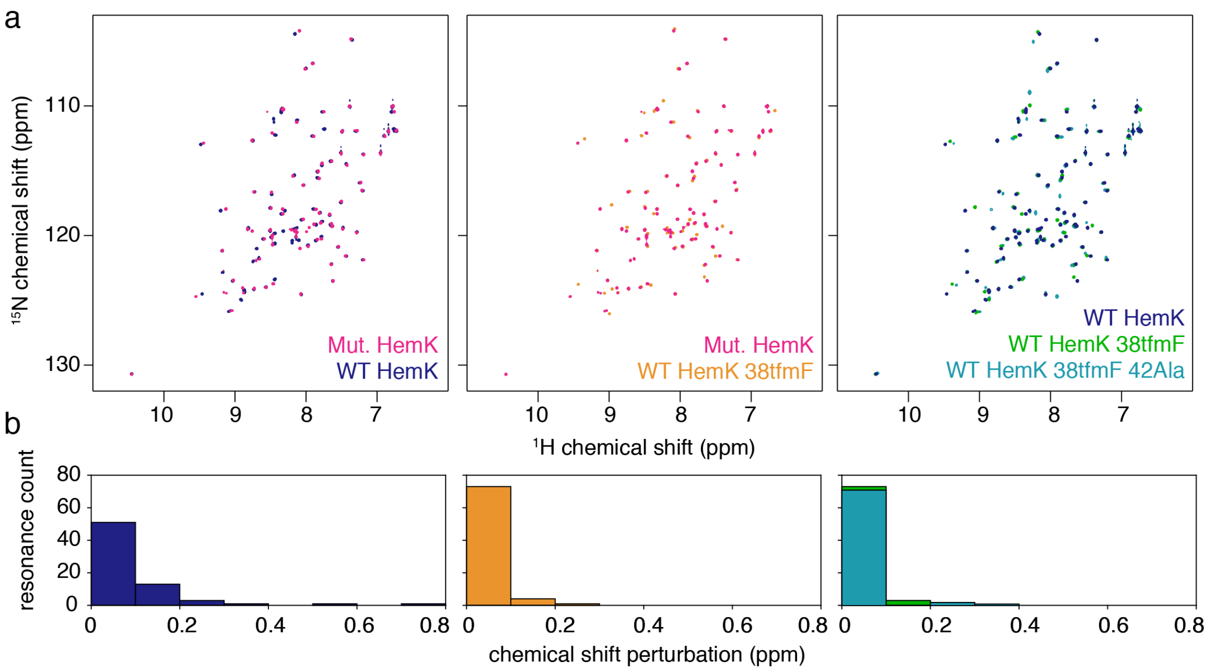

Supplementary Figure 7. NMR characterisation of the HemK NTD. **a.** 2D ^{1}H,^{15}N-HSQC spectra of HemK NTD variants, recorded at 298 K and 800 MHz. **b.** Histograms of chemical shift perturbations (CSP = $[(\Delta\delta_H)^2 + (0.2 * \Delta\delta_N)^2]^{1/2}$, where $\Delta\delta_H$ and $\Delta\delta_N$ are differences in the ^{1}H and ^{15}N chemical shifts respectively) from analysis of spectra shown in a.

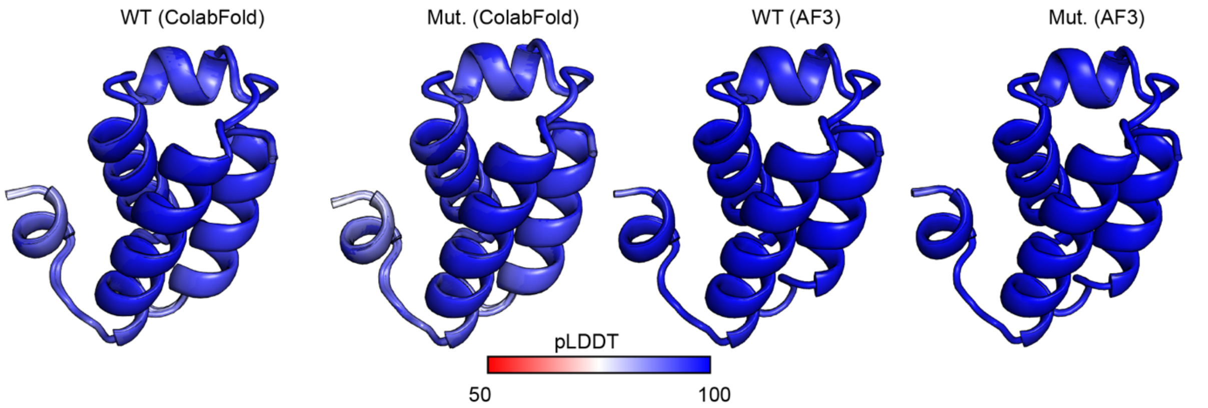

Supplementary Figure 8. ColabFold and Alphafold3 (AF3) predictions of wild-type (WT) and mutant (Mut.) HemK coloured according to the pLDDT score. Default settings were used without templates for ColabFold.

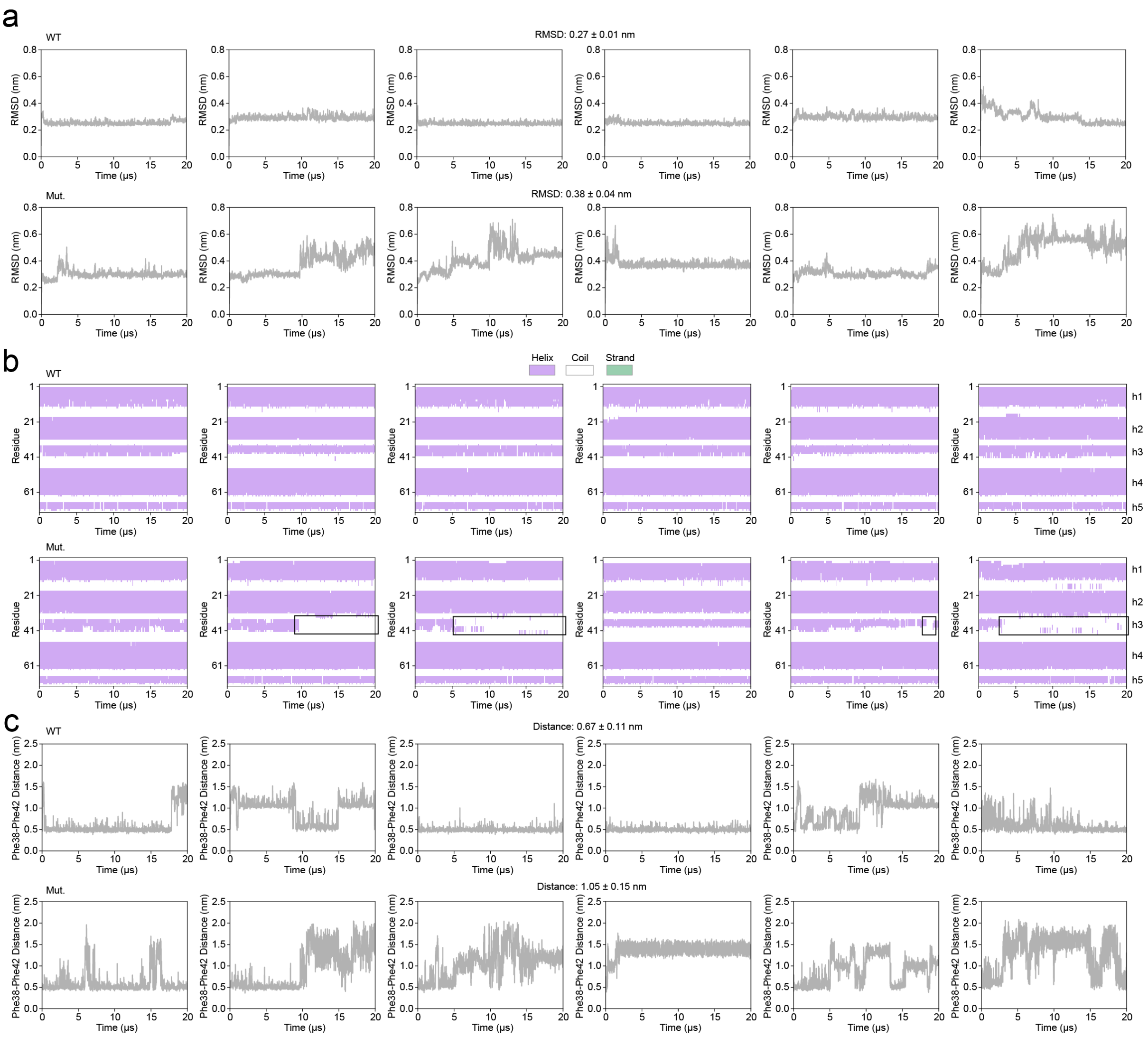

Supplementary Figure 9. Long-timescale, all-atom MD simulations of HemK (residues 1-73, NTD). **a.** All-atom protein RMSD relative to the crystal structure (residues 1-73, PDB 1T43) of six independent MD simulations of 20 μs per variant. **b.** Secondary structure content (calculated with DSSP) of HemK

observed during the MD simulations. The black boxes indicate parts of trajectories where helix h3 has completely unfolded for at least 1 μs. **c.** Distance between the centres of mass of the Phe38 and Phe42 aromatic rings. All averages in this figure represent the mean ± s.e.m.

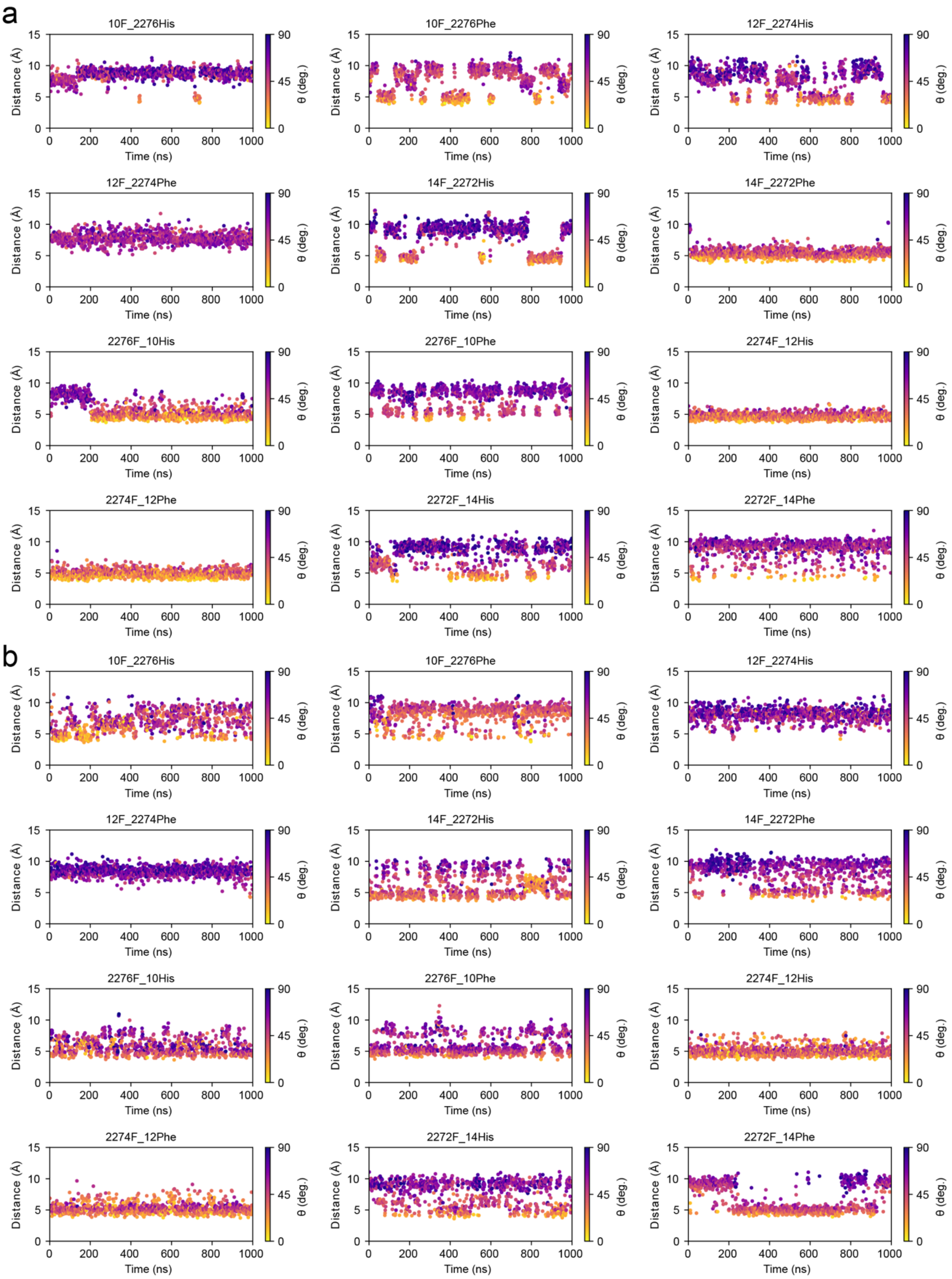

Supplementary Figure 10. Using all-atom MD simulations of the FLNa21-migfilin complex to screen suitable fluorine labelling sites for protein-protein interaction detection with the **a.** ff15ipq and **b**. C36m force field.

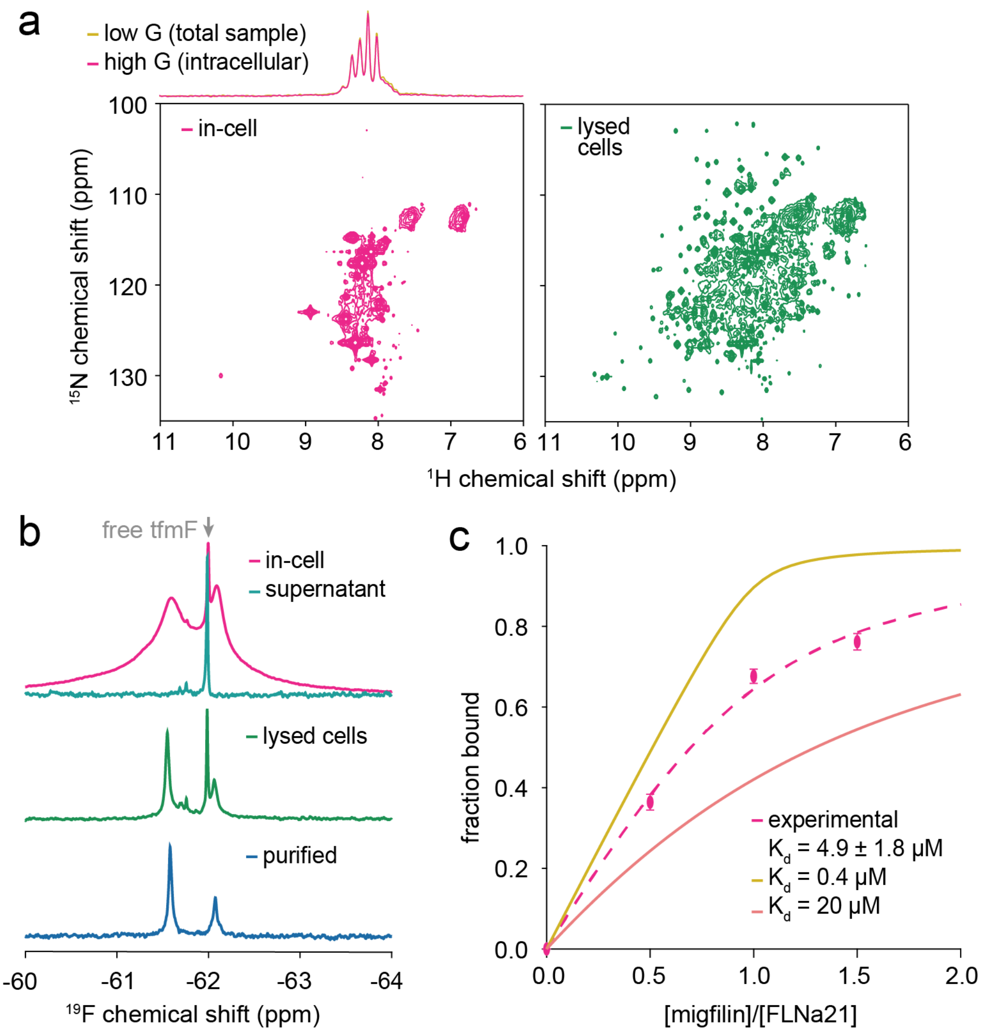

Supplementary Figure 11. Characterisation of FLNa21-migfilin binding by NMR in living cells and buffer. **a.** In-cell and lysate 2D ^{1}H,^{15}N SOFAST-HMQC spectra of FLNa21 recorded at 298 K and 800 MHz. Top shows ^{1}H,^{15}N-SORDID diffusion measurements to detect cell leakage (see Methods). **b.** ^{19}F NMR spectra of in-cell, lysate and purified FLNa21 2274tfmF co-expressed with migfilin 12His, including the supernatant obtained after centrifugation of the in-cell sample at the end of data acquisition. **c.** Titration and binding affinity fitting of migfilin 12His binding to FLNa21 2274tfmF (25 μM) under purified conditions (spectra shown in Fig. 5e). Theoretical binding curves of an upper[1] and lower[2] bound binding affinities from the literature are shown as a comparison.

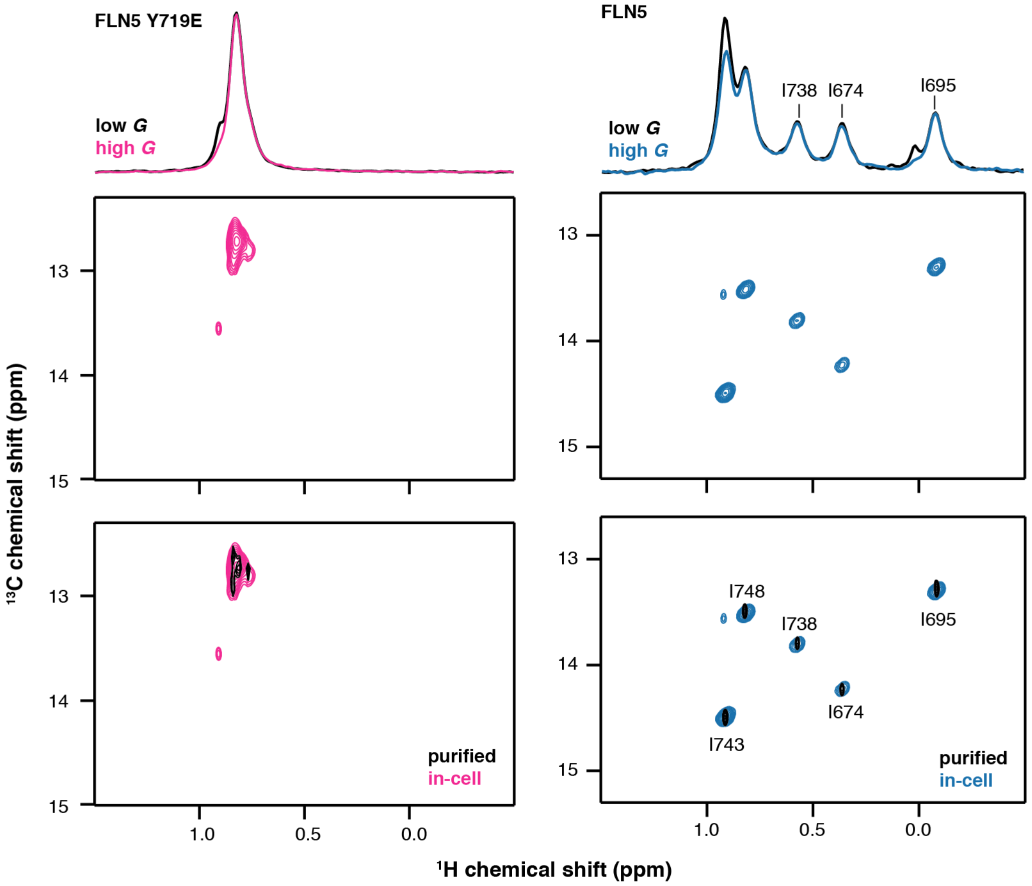

Supplementary Figure 12. ^{1}H,^{13}C HMQC NMR spectra of uniform ^{2}H, selectively Ileδ1-[$^{13}CH_3$]-labelled unfolded (left) FLN5 and folded (right) FLN5 recorded at 298K and 500 MHz. Top shows 1D ^{1}H,^{13}C diffusion measurements to detect cell leakage (see Methods).

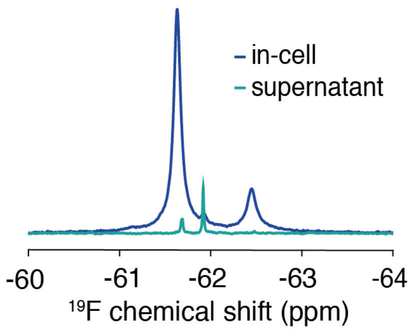

Supplementary Figure 13. Quality control for in-cell NMR of FLN5 672A. Overlaid ^{19}F NMR spectra of in-cell FLN5 672A and the supernatant after centrifugation of the in-cell sample at the end of NMR acquisition recorded at 298 K and 500 MHz.

Supplementary tables

Atom name	GAFF2 atom type	Charge
PA	p5	1.856920
PB	p5	2.238590
C5’	c3	0.133400
O5’	os	-0.607690
C4’	c3	0.260670
O4’	os	-0.514220
C3’	c3	0.269080
O3’	oh	-0.746930
C2’	c3	0.215010
O2’	oh	-0.746930
C1’	c3	0.269860
N1	ns	-0.763260
O1A	o	-1.063290
O1B	o	-1.237150
C2	cc	0.896060
N2	nv	-0.950460
O2A	o	-1.063290
O2B	o	-1.237150
N3	nd	-0.770020
O3A	os	-0.793990
O3B	o	-1.237150
C4	cd	0.397790
C5	cc	-0.018970
C6	c	0.866040
O6	o	-0.785660
N7	nc	-0.713190
C8	cd	0.300380
N9	na	-0.167520
H1	hn	0.462020
H8	h5	0.155810
H1’	h2	0.098850
H2’	ho	0.470330
H21	hn	0.422320
H3’	h1	0.028270
H22	hn	0.430200
H4’	h1	0.073600
H5’	h1	0.032150
H3T	ho	0.470330
H2’’	h1	0.037040
H5’’	h1	0.032150

Supplementary Table 1. GDP partial charges used in this work (obtained with the IPolQ method).

Protein	Variant	$\Delta G_{folding}$ (kcal mol^{-1})
FLN5	Wild-type	-7.01 ± 0.22
FLN5	655F	-6.58 ± 0.25
FLN5	673F 716His	-7.14 ± 0.28
FLN5	675F	-7.22 ± 0.26
FLN5	694F	-7.14 ± 0.26
FLN5	718F 671Phe	-7.30 ± 0.25
FLN5	718F 706His	-6.35 ± 0.25
FLN5	726F 746Phe	-7.30 ± 0.25
FLN5	728F 744His	-6.44 ± 0.26
FLN5	732F	-5.81 ± 0.26
HRAS (1-166)	Wild-type	-5.40 ± 0.10
HRAS (1-166)	32F	-4.58 ± 0.15
HRAS (1-166)	137F	-5.42 ± 0.17
HRAS (1-166)	157F 153His	-3.45 ± 0.11

Supplementary Table 2. Folding free energies ($\Delta G_{folding}$) of fluorinated and wild-type proteins. The free energies were determined from the populations of unfolded and folded protein in urea relative to wild-type as measured by ^{19}F NMR. Wild-type values for FLN5 and HRAS were previously measured[3,4,5]. "F" stands for 4-trifluoromethyl-L-phenylalanine (fluorine labelling site).

Protein	Variant	Chemical shift (ppm)	Secondary shift (ppm)	r (Å)	r SD	% inplane	% inplane SD	tfmF pLDDT	Aromatic pLDDT	Geom.	Geom. SD
FLN5	655F	-62.61	-0.79	4.22	0.1	0	0	98.82	98.74	-1.62E+22	2.03E+21
FLN5	665F_748Phe	-61.68	0.14	6.04	0.14	0	0	98.28	98.1	-4.32E+21	3.81E+20
FLN5	673F_716His	-62.17	-0.35	3.84	0.08	0	0	98.84	98.84	-2.88E+22	3.26E+21
FLN5	673F_716Phe	-62.09	-0.27	4.24	0.05	0	0	98.88	98.84	-1.08E+22	2.69E+21
FLN5	675F	-61.67	0.15	7.1	0.06	100	0	98.72	98.82	5.40E+20	1.43E+20
FLN5	675F_714Phe	-61.69	0.13	6.24	0.1	0	0	98.58	98.22	-3.70E+21	1.77E+21
FLN5	692F_732Phe	-61.69	0.13	7.44	0.05	0	0	98.16	98.72	-7.89E+20	6.02E+19
FLN5	694F_730Phe	-61.69	0.13	8.18	0.77	20	40	98.5	98.6	-6.47E+20	4.73E+20
FLN5	694F_730His	-61.58	0.24	8	0.06	0	0	98.56	98.64	-2.80E+21	2.48E+20
FLN5	696F_728Phe	-61.96	-0.14	8.06	0.14	100	0	98.52	98.3	1.08E+21	1.34E+20
FLN5	710F_716His	-62.1	-0.28	3.98	0.23	0	0	98.32	98.82	-1.66E+22	5.14E+21
FLN5	714F	-61.63	0.19	6.38	0.12	100	0	98.26	98.6	3.43E+21	1.17E+20
FLN5	716F_673Phe	-61.51	0.31	6.88	0.1	0	0	98.84	98.88	-2.17E+21	1.30E+20
FLN5	718F_671Phe	-62.29	-0.47	5.72	2.15	40	48.99	98.26	98.6	-1.26E+22	1.18E+22
FLN5	718F_706His	-62.21	-0.39	3.4	0.24	0	0	97.96	98.3	-4.44E+22	9.60E+21
FLN5	718F_706Phe	-61.91	-0.09	5.04	1.85	0	0	97.78	98.14	-2.27E+22	1.84E+22
FLN5	726F_746Phe	-62.52	-0.7	5.08	0.04	0	0	98.02	97.9	-2.85E+21	6.66E+20
FLN5	728F_744His	-62.39	-0.57	4.84	0.9	60	48.99	98.14	97.86	2.91E+21	5.46E+21
FLN5	728F_744Phe	-61.79	0.03	5.7	1.19	40	48.99	98.04	97.6	1.71E+21	6.46E+21
FLN5	732F_692Trp	-61.7	0.12	7.36	0.73	100	0	98.7	97.86	1.41E+21	3.35E+20
FLN5	732F_692Phe	-61.69	0.13	7.68	0.1	100	0	98.76	98.4	2.09E+21	9.49E+19
FLN5	732F_692His	-61.62	0.2	7.78	0.04	100	0	98.74	98.36	1.90E+21	5.13E+19
FLN5	740F	-62.06	-0.24	7.82	0.17	0	0	98.12	98.84	-2.16E+21	1.97E+20
FLN5	748F	-61.85	-0.03	8.46	0.05	0	0	98.1	98.22	-2.12E+21	2.47E+20
FLN4	555F	-62.81	-0.99	4.14	0.19	0	0	86.5	86.78	-2.05E+22	3.76E+21
FLN4	565F_646His	-62.05	-0.23	6.28	0.36	20	40	86.38	89.38	-9.47E+20	8.69E+20
FLN4	616F_571His	-62.08	-0.26	5.7	0.17	0	0	91.34	89.62	-6.79E+21	8.47E+20
FLN4	624F_644Phe	-62.52	-0.7	4.78	0.1	0	0	92.8	91.62	-8.87E+21	1.33E+21
I27	5F_24Phe	-61.71	0.11	10.84	0.83	80	40	95.02	97.94	-2.06E+20	8.66E+20
I27	6F_24Phe	-61.84	-0.02	7.02	0.07	0	0	97.94	98.28	-5.47E+21	2.33E+20
I27	9F_22Phe	-61.66	0.16	9.26	0.59	80	40	98.18	97.92	7.44E+20	7.22E+20
I27	14F_87Phe	-62	-0.18	6.2	0.13	0	0	98.38	98.14	-5.09E+21	5.62E+20
I27	14F_87His	-62.15	-0.33	5.9	0.06	0	0	98.42	98.32	-5.47E+21	5.26E+20
I27	20F_61Trp	-61.73	0.09	11.54	0.08	0	0	98.7	98.58	-4.55E+20	1.86E+19
I27	59F	-62.18	-0.36	3.94	0.14	0	0	98.7	98.5	-2.26E+22	7.74E+21
I27	72F_35His	-62.17	-0.35	3.24	0.08	0	0	98.68	98.74	-4.74E+22	4.77E+21
FLNa21	2242F	-62.2	-0.38	7.18	0.04	0	0	95.62	95.04	-2.33E+20	1.02E+20
FLNa21	2244F	-61.11	0.71	7.3	2.88	100	0	96.82	94.34	2.90E+21	3.57E+21
FLNa21	2258F_2296Phe	-62.1	-0.28	7.36	1.2	20	40	97.9	98.08	-2.15E+21	1.88E+21
FLNa21	2306F_2322His	-62.53	-0.71	4.74	0.05	0	0	98.6	98.54	-9.27E+21	2.23E+21
HemK	38F	-62.04	-0.22	4.74	0.08	0	0	93.4	94.34	-1.40E+22	1.49E+21
HRAS	5F_54His	-62.08	-0.26	4.06	0.12	0	0	98	97.86	-2.32E+22	3.34E+21
HRAS	32F	-61.37	0.45	13.56	2.98	100	0	66.26	93.28	5.49E+20	6.92E+20
HRAS	88F_92His	-62.28	-0.46	3.64	0.08	20	40	96.46	94.34	-1.48E+22	1.02E+22
HRAS	99F_103His	-61.73	0.09	4.1	0.17	20	40	91.44	91.4	-7.31E+21	6.13E+21
HRAS	137F	-61.13	0.69	4.14	0.05	100	0	97.58	96.18	1.22E+22	2.18E+21
HRAS	157F_153His	-62.33	-0.51	4.34	0.2	0	0	98.52	97.94	-2.16E+22	3.57E+21
FLNa21-Mig	2274F	-61.55	0.03	5.46	1.3	100	0	97.2	94.46	5.13E+21	2.39E+21
FLNa21-Mig	2274F_12His	-62.07	-0.49	6.06	0.23	0	0	97.2	95.34	-5.83E+21	8.07E+20

Supplementary Table 3. ^{19}F NMR chemical shifts and descriptors calculated from ColabFold predictions. Geometric factors (Geom.) are given in cm^{-3}. HRAS 28F (28tfmF) was not predicted because AF2/ColabFold does not model ligands. “F” stands for 4-trifluoromethyl-L-phenylalanine (fluorine labelling site).

Protein	Variant	Chemical shift (ppm)	Secondary shift (ppm)	r (Å)	r SD	% inplane	% inplane SD	tfmF pLDDT	Aromatic pLDDT	Geom.	Geom. SD
FLN5	655F	-62.61	-0.79	4.18	0.04	0	0	97.26	98.8	-1.96E+22	7.03E+20
FLN5	665F_748Phe	-61.68	0.14	6.06	0.05	0	0	95.4	97.66	-4.08E+21	1.90E+20
FLN5	673F_716His	-62.17	-0.35	4	0	0	0	94.84	97.8	-2.31E+22	8.49E+20
FLN5	673F_716Phe	-62.09	-0.27	4.46	0.08	0	0	96.28	98.5	-6.85E+21	2.30E+21
FLN5	675F	-61.67	0.15	7.06	0.05	100	0	97.94	98.42	5.81E+20	4.19E+19
FLN5	675F_714Phe	-61.69	0.13	6.12	0.04	0	0	96.58	96.02	-6.88E+21	6.46E+20
FLN5	692F_732Phe	-61.69	0.13	6.52	1.56	0	0	69.16	98.58	-8.96E+21	1.72E+22
FLN5	694F_730Phe	-61.69	0.13	7.9	0	0	0	91.42	93.9	-2.39E+20	6.41E+19
FLN5	694F_730His	-61.58	0.24	7.94	0.05	0	0	87.94	93.12	-2.69E+21	7.02E+19
FLN5	696F_728Phe	-61.96	-0.14	7.78	0.04	100	0	88.94	89.4	1.37E+21	4.33E+19
FLN5	710F_716His	-62.1	-0.28	4.14	0.05	0	0	92.78	95.24	-1.35E+22	3.78E+20
FLN5	714F	-61.63	0.19	6.12	0.04	100	0	78.48	98.44	3.88E+21	2.16E+20
FLN5	716F_673Phe	-61.51	0.31	7.06	0.08	0	0	95.16	98.4	-2.44E+21	9.53E+19
FLN5	718F_671Phe	-62.29	-0.47	5.04	1.16	0	0	56.1	89.18	-9.92E+21	6.28E+21
FLN5	718F_706His	-62.21	-0.39	7.3	2.28	60	48.99	53.3	87.52	-6.52E+21	1.40E+22
FLN5	718F_706Phe	-61.91	-0.09	8.76	1.75	80	40	54.16	89.84	1.49E+20	1.08E+20
FLN5	726F_746Phe	-62.52	-0.7	5	0	0	0	90.94	96.72	-7.16E+21	5.91E+20
FLN5	728F_744His	-62.39	-0.57	6.66	0.83	20	40	61.52	89.74	-1.22E+21	2.53E+21
FLN5	728F_744Phe	-61.79	0.03	7.44	0.14	0	0	81.6	93.64	-2.80E+21	8.95E+19
FLN5	732F_692Trp	-61.7	0.12	6.38	0.26	60	48.99	96.32	89.32	4.14E+20	7.23E+20
FLN5	732F_692Phe	-61.69	0.13	7.58	0.04	100	0	96.34	96.5	2.22E+21	3.59E+19
FLN5	732F_692His	-61.62	0.2	7.68	0.04	100	0	95.72	92.12	1.82E+21	7.01E+19
FLN5	740F	-62.06	-0.24	8.06	0.08	0	0	86.9	98.48	-2.02E+21	8.65E+19
FLN5	748F	-61.85	-0.03	8.5	0	0	0	94.5	97.9	-1.72E+21	3.58E+19
FLN4	555F	-62.81	-0.99	3.82	0.04	0	0	97.24	98.54	-3.12E+22	1.04E+21
FLN4	565F_646His	-62.05	-0.23	5.5	0.09	0	0	93.98	97.14	-3.39E+21	4.31E+20
FLN4	616F_571His	-62.08	-0.26	6.08	0.04	0	0	95.92	91.56	-4.63E+21	1.43E+20
FLN4	624F_644Phe	-62.52	-0.7	5.1	0	0	0	94.64	97.98	-6.30E+21	2.09E+20
I27	5F_24Phe	-61.71	0.11	10.46	1.04	100	0	70.74	89.96	7.23E+20	4.68E+20
I27	6F_24Phe	-61.84	-0.02	7.2	0.35	20	40	63.9	94.16	-4.14E+21	2.59E+21
I27	9F_22Phe	-61.66	0.16	9.04	0.85	60	48.99	80.5	87.38	4.73E+20	7.50E+20
I27	14F_87Phe	-62	-0.18	6.16	0.05	0	0	93.34	96.92	-3.79E+21	1.85E+20
I27	14F_87His	-62.15	-0.33	5.64	0.1	0	0	93.28	96.76	-7.87E+21	1.18E+21
I27	20F_61Trp	-61.73	0.09	10.32	2.46	0	0	75.62	96.46	-4.29E+20	8.30E+19
I27	59F	-62.18	-0.36	4.14	0.05	0	0	95.54	96.06	-2.08E+22	1.87E+21
I27	72F_35His	-62.17	-0.35	3.48	0.04	0	0	91.88	97.72	-3.68E+22	1.94E+21
FLNa21	2242F	-62.2	-0.38	7.6	2.05	40	48.99	56.94	93.16	-2.17E+21	4.43E+21
FLNa21	2244F	-61.11	0.71	9.3	1.41	40	48.99	63.8	90.74	-2.01E+20	6.51E+20
FLNa21	2258F_2296Phe	-62.1	-0.28	6.74	1.61	40	48.99	57.38	92.32	-5.50E+21	6.28E+21
FLNa21	2306F_2322His	-62.53	-0.71	4.6	0.18	0	0	91.58	96.84	-8.70E+21	3.74E+21
HemK	38F	-62.04	-0.22	5	0	0	0	95.06	96.98	-9.82E+21	1.47E+20
HRAS	5F_54His	-62.08	-0.26	3.9	0.13	0	0	89.82	95.52	-2.62E+22	2.74E+21
HRAS	28F	-62.24	-0.46	3.54	0.05	0	0	93.94	98.36	-2.33E+22	1.70E+21
HRAS	32F	-61.37	0.45	7.52	3.89	100	0	51.96	93.98	4.65E+21	2.26E+21
HRAS	88F_92His	-62.28	-0.46	4.06	0.37	0	0	84.76	88.08	-1.84E+22	1.31E+22
HRAS	99F_103His	-61.73	0.09	3.88	0.04	0	0	72.1	86.92	-1.60E+22	3.20E+21
HRAS	137F	-61.13	0.69	4.36	0.87	100	0	95.08	91.38	1.40E+22	4.89E+21
HRAS	157F_153His	-62.33	-0.51	4.84	0.15	100	0	87.36	89.76	8.67E+21	7.44E+20
FLNa21-Mig	2274F	-61.55	0.03	6.12	1.25	100	0	88.72	91.98	3.36E+21	1.86E+21
FLNa21-Mig	2274F_12His	-62.07	-0.49	5.22	0.29	0	0	89.5	94.24	-9.85E+21	2.85E+21

Supplementary Table 4. ^{19}F NMR chemical shifts and descriptors calculated from AF3 predictions. Geometric factors (Geom.) are given in cm^{-3}. "F" stands for 4-trifluoromethyl-L-phenylalanine (fluorine labelling site).

Protein	Variant	Chemical shift (ppm)	Secondary shift (ppm)	r (Å)	r SEM	% inplane	% inplane SEM	Geom.	Geom. SEM
FLN5	655F	-62.61	-0.79	4.69	0.02	0.2	0.12	-1.28E+22	3.23E+20
FLN5	665F_748Phe	-61.68	0.14	5.53	0.01	13.23	0.78	-7.18E+21	2.03E+20
FLN5	673F_716His	-62.17	-0.35	5.06	0.02	18.07	0.9	-7.25E+21	3.79E+20
FLN5	673F_716Phe	-62.09	-0.27	4.95	0.01	5.1	0.57	-9.83E+21	4.78E+20
FLN5	675F	-61.67	0.15	7.73	0.02	81.77	0.67	6.60E+20	9.62E+18
FLN5	675F_714Phe	-61.69	0.13	6.93	0.53	48.07	20.67	-2.23E+21	1.59E+21
FLN5	692F_732Phe	-61.69	0.13	8.6	0.4	55.57	4.37	-7.22E+20	3.14E+20
FLN5	694F_730Phe	-61.69	0.13	7.7	0.4	53.17	8.95	-2.76E+21	1.39E+21
FLN5	694F_730His	-61.58	0.24	8.89	0.13	73.67	8.61	5.39E+20	1.78E+20
FLN5	696F_728Phe	-61.96	-0.14	5.85	0.18	9.5	4.14	-6.23E+21	3.73E+20
FLN5	710F_716His	-62.1	-0.28	6.17	0.23	28.7	3.63	-4.78E+21	9.01E+20
FLN5	714F	-61.63	0.19	8.42	0.15	57.93	11.94	3.93E+20	3.12E+20
FLN5	716F_673Phe	-61.51	0.31	7.74	0.16	36.43	4.76	-9.50E+20	2.49E+20
FLN5	718F_671Phe	-62.29	-0.47	5.3	0.12	17	3.49	-6.90E+21	5.75E+20
FLN5	718F_706His	-62.21	-0.39	8.24	0.69	57.23	4.88	-2.49E+21	1.24E+21
FLN5	718F_706Phe	-61.91	-0.09	8.04	0.39	62.03	8.25	-1.35E+21	1.34E+21
FLN5	726F_746Phe	-62.52	-0.7	5.48	0.19	14.6	1.91	-9.14E+21	6.70E+20
FLN5	728F_744His	-62.39	-0.57	5.87	0.76	26.7	8.49	-6.13E+21	1.54E+21
FLN5	728F_744Phe	-61.79	0.03	7.28	0.47	51.7	4.36	-1.29E+21	7.51E+20
FLN5	732F_692Trp	-61.7	0.12	6.8	0.39	59.57	7.24	-1.44E+20	2.68E+20
FLN5	732F_692Phe	-61.69	0.13	6.29	0.09	43.67	1.9	-9.36E+20	7.66E+19
FLN5	732F_692His	-61.62	0.2	6.62	0.29	59.37	4.1	-2.46E+20	1.51E+20
FLN5	740F	-62.06	-0.24	9.45	0.12	4.4	0.61	-1.43E+21	8.51E+19
FLN5	748F	-61.85	-0.03	8.62	0.15	9.9	0.31	-1.17E+21	8.62E+18
FLN4	555F	-62.81	-0.99	4.31	0.03	0.03	0.03	-2.11E+22	8.62E+20
FLN4	565F_646His	-62.05	-0.23	6.02	0.57	22.4	6.37	-6.98E+21	8.74E+20
FLN4	616F_571His	-62.08	-0.26	5.07	0.01	8.17	0.33	-7.20E+21	1.74E+20
FLN4	624F_644Phe	-62.52	-0.7	5.12	0.02	3.87	0.24	-1.02E+22	1.27E+20
I27	5F_24Phe	-61.71	0.11	9.77	0.83	63.57	14.92	-5.41E+20	4.61E+20
I27	6F_24Phe	-61.84	-0.02	7.9	0.49	35.87	7.83	-1.04E+21	4.71E+20
I27	9F_22Phe	-61.66	0.16	10.1	0.14	58.83	5.41	2.54E+20	5.93E+19
I27	14F_87Phe	-62	-0.18	6.77	0.82	19.7	11.36	-4.58E+21	1.81E+21
I27	14F_87His	-62.15	-0.33	5.58	0.02	6.07	0.47	-8.55E+21	3.69E+19
I27	20F_61Trp	-61.73	0.09	7.1	0.04	87.7	2.28	1.66E+21	1.21E+20
I27	59F	-62.18	-0.36	5.4	0.23	12.23	2.4	-8.52E+21	9.73E+20
I27	72F_35His	-62.17	-0.35	6.65	0.67	9.9	2.26	-9.77E+21	3.02E+21
FLNa21	2242F	-62.2	-0.38	5.57	0.03	7.83	0.91	-3.84E+21	3.38E+19
FLNa21	2244F	-61.11	0.71	6.63	0.26	55.23	12.43	-3.83E+21	2.73E+21
FLNa21	2258F_2296Phe	-62.1	-0.28	5.47	0.15	5.7	2.33	-1.16E+22	5.01E+20
FLNa21	2306F_2322His	-62.53	-0.71	4.79	0.02	5.27	0.15	-1.14E+22	1.54E+20
HemK	38F	-62.04	-0.22	5.14	0.02	1.13	0.44	-9.79E+21	4.77E+20
HRAS	5F_54His	-62.08	-0.26	4.56	0.02	0.8	0.12	-1.41E+22	4.24E+20
HRAS	28F	-62.24	-0.46	4.41	0.07	0.13	0.11	-1.94E+22	2.40E+20
HRAS	32F	-61.37	0.45	6.32	0.45	83.57	11.47	7.06E+20	1.65E+21
HRAS	88F_92His	-62.28	-0.46	11.25	0.11	89.33	1.47	5.07E+20	1.16E+20
HRAS	99F_103His	-61.73	0.09	6.17	0.33	28.53	3.65	-2.89E+21	5.00E+20
HRAS	137F	-61.13	0.69	6.73	0.17	66.53	4.45	-4.68E+20	9.00E+20
HRAS	157F_153His	-62.33	-0.51	5.4	0.09	53.1	9.44	-1.98E+21	1.33E+21
FLNa21-Mig	2274F	-61.55	0.03	8.04	1.37	56.37	3.7	-4.36E+19	1.24E+20
FLNa21-Mig	2274F_12His	-62.07	-0.49	4.75	0.01	6.1	0.79	-1.08E+22	2.66E+19

Supplementary Table 5. ^{19}F NMR chemical shifts and descriptors calculated from MD (ff15ipq) predictions. Geometric factors (Geom.) are given in cm^{-3}. “F” stands for 4-trifluoromethyl-L-phenylalanine (fluorine labelling site).

Protein	Variant	Chemical shift (ppm)	Secondary shift (ppm)	r (Å)	r SEM	% inplane	% inplane SEM	Geom.	Geom. SEM
FLN5	655F	-62.61	-0.79	4.83	0.01	0.03	0.03	-1.22E+22	1.63E+20
FLN5	665F_748Phe	-61.68	0.14	6.89	0.24	40.27	2.92	-2.17E+21	2.94E+20
FLN5	673F_716His	-62.17	-0.35	5.21	0.05	33.3	1.12	-3.91E+21	1.57E+20
FLN5	673F_716Phe	-62.09	-0.27	5.31	0.17	9.7	2.92	-8.39E+21	4.82E+20
FLN5	675F	-61.67	0.15	7.7	0.01	66.27	2.38	2.89E+20	5.63E+19
FLN5	675F_714Phe	-61.69	0.13	6.31	0.05	23.47	9.08	-4.04E+21	9.72E+20
FLN5	692F_732Phe	-61.69	0.13	9.38	0.09	45.03	0.81	-1.28E+20	1.71E+19
FLN5	694F_730Phe	-61.69	0.13	8.9	0.03	76	0.37	5.83E+20	2.32E+19
FLN5	694F_730His	-61.58	0.24	9.14	0.02	77.83	0.82	5.33E+20	1.39E+19
FLN5	696F_728Phe	-61.96	-0.14	9.68	0.19	72.83	3.1	7.05E+18	2.07E+20
FLN5	710F_716His	-62.1	-0.28	6.04	0.07	6.23	0.81	-6.96E+21	1.68E+20
FLN5	714F	-61.63	0.19	8.71	0.02	93.93	0.78	8.90E+20	1.56E+19
FLN5	716F_673Phe	-61.51	0.31	6.07	0.14	5.97	1.79	-7.31E+21	7.39E+20
FLN5	718F_671Phe	-62.29	-0.47	5.41	0.18	15.73	3.35	-8.85E+21	5.80E+20
FLN5	718F_706His	-62.21	-0.39	6.76	0.61	38.27	9.87	-5.12E+21	1.41E+21
FLN5	718F_706Phe	-61.91	-0.09	7.11	0.29	45.77	6.6	-2.49E+21	7.20E+20
FLN5	726F_746Phe	-62.52	-0.7	5	0	4.77	0.46	-1.12E+22	1.05E+20
FLN5	728F_744His	-62.39	-0.57	5.38	0.06	20.9	0.47	-7.40E+21	1.51E+20
FLN5	728F_744Phe	-61.79	0.03	5.16	0.03	20.67	0.71	-7.06E+21	2.41E+20
FLN5	732F_692Trp	-61.7	0.12	7.49	0.19	71.23	5.58	3.82E+20	2.41E+20
FLN5	732F_692Phe	-61.69	0.13	6.21	0.36	40.77	7.44	-9.73E+20	3.28E+20
FLN5	732F_692His	-61.62	0.2	5.6	0	32.7	0.69	-2.05E+21	1.87E+19
FLN5	740F	-62.06	-0.24	9.52	0.07	10.2	1.01	-1.26E+21	3.29E+19
FLN5	748F	-61.85	-0.03	9.55	0.02	21.87	3.49	-6.27E+20	6.15E+19
FLN4	555F	-62.81	-0.99	5.7	0.46	13.4	5.45	-1.35E+22	1.20E+21
FLN4	565F_646His	-62.05	-0.23	5.28	0.01	10.27	0.32	-8.15E+21	1.03E+20
FLN4	616F_571His	-62.08	-0.26	5.4	0.14	8.53	1.53	-8.59E+21	4.12E+20
FLN4	624F_644Phe	-62.52	-0.7	5.14	0.01	2.53	0.26	-1.02E+22	1.64E+20
I27	5F_24Phe	-61.71	0.11	8.54	0.27	31.37	9.53	-1.29E+21	3.30E+20
I27	6F_24Phe	-61.84	-0.02	7.84	0.08	21.2	5.75	-2.64E+21	3.66E+20
I27	9F_22Phe	-61.66	0.16	10.26	0.77	68.37	1.38	6.76E+20	2.18E+20
I27	14F_87Phe	-62	-0.18	6.22	0.02	16.53	0.56	-3.75E+21	1.20E+20
I27	14F_87His	-62.15	-0.33	6.07	0.02	3.2	1	-6.95E+21	4.94E+19
I27	20F_61Trp	-61.73	0.09	6.88	0.43	68.27	6.81	4.16E+20	1.81E+20
I27	59F	-62.18	-0.36	5.34	0.07	17.1	1.68	-6.93E+21	2.73E+20
I27	72F_35His	-62.17	-0.35	5.86	0.35	22.5	8.75	-5.13E+21	8.25E+20
FLNa21	2242F	-62.2	-0.38	6.58	0.58	15.53	5.9	-5.51E+21	8.72E+20
FLNa21	2244F	-61.11	0.71	6.27	0.35	44.6	13.84	-2.69E+21	2.00E+21
FLNa21	2258F_2296Phe	-62.1	-0.28	5.63	0.11	21.43	1.74	-8.08E+21	4.13E+20
FLNa21	2306F_2322His	-62.53	-0.71	4.78	0.01	4.07	0.4	-1.16E+22	2.22E+20
FLNa21-Mig	2274F	-61.55	0.03	7.4	0.06	63.4	2.29	5.65E+20	9.10E+19
FLNa21-Mig	2274F_12His	-62.07	-0.49	5.09	0.02	12.5	0.69	-7.58E+21	4.67E+19

Supplementary Table 6. ^{19}F NMR chemical shifts and descriptors calculated from MD (C36m) predictions. Geometric factors (Geom.) are given in cm^{-3}. HemK and HRAS variants were not predicted with C36m due to protein instability observed for these wild-type proteins with C36m. "F" stands for 4-trifluoromethyl-L-phenylalanine (fluorine labelling site).

Full dataset					C36m dataset				
	ColabFold	AF3	ff15ipq	Combined		ColabFold	AF3	ff15ipq	C36m
**Total variants**	49	50	50	50	**Total variants**	42	42	42	42
**Predicted positives**	20	22	21	31	**Predicted positives**	14	15	17	15
**Predicted negatives**	29	28	29	19	**Predicted negatives**	28	27	25	18
**Actual positives**	29	30	30	30	**Actual positives**	23	23	23	23
**Actual negatives**	20	20	20	20	**Actual negatives**	19	19	19	19
**TN**	16	18	19	25	**TN**	11	13	15	14
**TP**	16	17	18	14	**TP**	16	17	17	18
**FP**	4	4	2	6	**FP**	3	2	2	1
**FN**	13	11	11	5	**FN**	12	10	8	9
**TPR**	0.55	0.62	0.63	0.83	**TPR**	0.48	0.57	0.65	0.61
**TNR**	0.8	0.81	0.9	0.7	**TNR**	0.84	0.89	0.89	0.95
**FPR**	0.2	0.19	0.1	0.3	**FPR**	0.16	0.11	0.11	0.05
**FNR**	0.45	0.38	0.37	0.17	**FNR**	0.52	0.43	0.35	0.39
**PPV**	0.8	0.82	0.9	0.81	**PPV**	0.79	0.87	0.88	0.93
**NPV**	0.55	0.61	0.62	0.74	**NPV**	0.57	0.63	0.68	0.67
**FDR**	0.2	0.18	0.1	0.19	**FDR**	0.21	0.13	0.12	0.07
**FOR**	0.45	0.39	0.38	0.26	**FOR**	0.43	0.37	0.32	0.33

Supplementary Table 7. Performance of predictive ^{19}F NMR ring current design strategy. TP = true positive; TN = true negative; FP = false positive; FN = false negative; TPR = true positive rate; FPR = false positive rate; TNR = true negative rate; FNR = false negative rate; PPV = positive predictive value; NPV = negative predictive value; FDR = false discovery rate; FOR = false omission rate. Positive and negative classifications and predictions are defined in the Methods section. The combined approach defines a positive prediction being predicted as a positive by at least one of the methods (ColabFold, AF3, ff15ipq MD) and a negative when predicted negative by all three methods.

		ColabFold	AF3
True predictions	**Mean**	97.3	87.7
	**S.D.**	2.5	11.3
	**Min.**	86.5	54.2
	**Max.**	98.8	97.9
	**pLDDT > 70 (%)**	100.0	88.9
	**pLDDT > 90 (%)**	96.9	61.1
False predictions	**Mean**	93.9	74.2
	**S.D.**	7.7	15.7
	**Min.**	66.3	52.0
	**Max.**	98.8	95.2
	**pLDDT > 70 (%)**	94.1	57.1
	**pLDDT > 90 (%)**	82.4	21.4

Supplementary Table 8. Minimum pLDDT of labelling pairs (tfmF/tyrosine and second aromatic) for true and false predictions. pLDDT values were averaged over all five predicted models.

Supplementary References

1 Pentikainen, U. *et al.* Assembly of a filamin four-domain fragment and the influence of splicing variant-1 on the structure. *J Biol Chem* **286**, 26921-26930, doi:10.1074/jbc.M110.195958 (2011).

2 Lad, Y. *et al.* Structural basis of the migfilin-filamin interaction and competition with integrin beta tails. *J Biol Chem* **283**, 35154-35163, doi:10.1074/jbc.M802592200 (2008).

3 Waudby, C. A. *et al.* Systematic mapping of free energy landscapes of a growing filamin domain during biosynthesis. *Proc Natl Acad Sci U S A* **115**, 9744-9749, doi:10.1073/pnas.1716252115 (2018).

4 Chan, S. H. S. *et al.* The ribosome stabilizes partially folded intermediates of a nascent multi-domain protein. *Nature Chemistry* **14**, 1165-1173, doi:10.1038/s41557-022-01004-0 (2022).

5 Streit, J. O. *et al.* The ribosome lowers the entropic penalty of protein folding. *Nature* **633**, 232-239, doi:10.1038/s41586-024-07784-4 (2024).
