# Supplementary material for: Rational design of 19F NMR labelling sites to probe protein structure and interactions
Source: Nat Commun. 2025 May 8;16:4300. doi: 10.1038/s41467-025-59105-6 (PMC12062419; doi:10.1038/s41467-025-59105-6)
Supplement: Supplementary file 2 — Reporting Summary [file 41467_2025_59105_MOESM2_ESM.pdf]

nature portfolio

Corresponding author(s): John Christodoulou

Last updated by author(s): Apr 8, 2025

Reporting Summary

Nature Portfolio wishes to improve the reproducibility of the work that we publish. This form provides structure for consistency and transparency in reporting. For further information on Nature Portfolio policies, see our Editorial Policies and the Editorial Policy Checklist.

Statistics

For all statistical analyses, confirm that the following items are present in the figure legend, table legend, main text, or Methods section.

n/a	Confirmed	
☐	☒	The exact sample size (*n*) for each experimental group/condition, given as a discrete number and unit of measurement
☐	☒	A statement on whether measurements were taken from distinct samples or whether the same sample was measured repeatedly
☒	☐	The statistical test(s) used AND whether they are one- or two-sided<br>*Only common tests should be described solely by name; describe more complex techniques in the Methods section.*
☒	☐	A description of all covariates tested
☒	☐	A description of any assumptions or corrections, such as tests of normality and adjustment for multiple comparisons
☐	☒	A full description of the statistical parameters including central tendency (e.g. means) or other basic estimates (e.g. regression coefficient) AND variation (e.g. standard deviation) or associated estimates of uncertainty (e.g. confidence intervals)
☒	☐	For null hypothesis testing, the test statistic (e.g. *F*, *t*, *r*) with confidence intervals, effect sizes, degrees of freedom and *P* value noted<br>*Give P values as exact values whenever suitable.*
☒	☐	For Bayesian analysis, information on the choice of priors and Markov chain Monte Carlo settings
☒	☐	For hierarchical and complex designs, identification of the appropriate level for tests and full reporting of outcomes
☐	☒	Estimates of effect sizes (e.g. Cohen's *d*, Pearson's *r*), indicating how they were calculated

Our web collection on statistics for biologists contains articles on many of the points above.

Software and code

Policy information about availability of computer code

Data collection	NMR data were recorded using Topspin 3.5pl2. Further details are provided in the Methods. MD simulations were performed and processed with GROMACS version 2021.3 and 2023.1. For model building and visualisation, PyMol version 2.3 was used.
Data analysis	NMR data were analysed using CCPN (version 2.4), nmrPipe (version 11.7) and MATLAB (R2017b, The MathWorks Inc.), codes are available on github.com/shschan/NMR-fit. Python analyses utilised version 3.7. Python scripts used to calculate ring current interactions are available on https://github.com/julian-streit/RingCurrents19F.

For manuscripts utilizing custom algorithms or software that are central to the research but not yet described in published literature, software must be made available to editors and reviewers. We strongly encourage code deposition in a community repository (e.g. GitHub). See the Nature Portfolio guidelines for submitting code & software for further information.

Data

Policy information about availability of data

All manuscripts must include a data availability statement. This statement should provide the following information, where applicable:

- Accession codes, unique identifiers, or web links for publicly available datasets
- A description of any restrictions on data availability
- For clinical datasets or third party data, please ensure that the statement adheres to our policy

All 19F NMR chemical shift data are available in the Supplementary Information. AlphaFold-generated structural models and MD trajectories are available on Zenodo at https://zenodo.org/records/14288915. Additional data are available as Supplementary Tables and as source data with the figures.

Research involving human participants, their data, or biological material

Policy information about studies with human participants or human data. See also policy information about sex, gender (identity/presentation), and sexual orientation and race, ethnicity and racism.

Reporting on sex and gender	n/a
Reporting on race, ethnicity, or other socially relevant groupings	n/a
Population characteristics	n/a
Recruitment	n/a
Ethics oversight	n/a

Note that full information on the approval of the study protocol must also be provided in the manuscript.

Field-specific reporting

Please select the one below that is the best fit for your research. If you are not sure, read the appropriate sections before making your selection.

☒ Life sciences ☐ Behavioural & social sciences ☐ Ecological, evolutionary & environmental sciences

For a reference copy of the document with all sections, see nature.com/documents/nr-reporting-summary-flat.pdf

Life sciences study design

All studies must disclose on these points even when the disclosure is negative.

Sample size	No samples sizes were predetermined. Samples sizes were chosen according to standards generally accepted in the protein folding/structural & computational biology fields. MD simulations were performed in triplicate (all tfmF variants compared to experimental chemical shifts) and six repeats were performed for long-timescale simulations of HemK.
Data exclusions	No data were excluded.
Replication	Independent MD replicates were used to assess uncertainty and reproducibility.
Randomization	Not applicable, as typical for NMR and structural biology studies. Experiments and simulations were rationally designed to be systematic and answer specific technical and biological questions and therefore randomization was not applicable. All experiments and simulations were performed under well-controlled conditions.
Blinding	Not applicable, as typical for NMR and structural biology studies. Our data analysis was systematic without any possible prior knowledge about the result and, thus, blinding was not applicable.

Reporting for specific materials, systems and methods

We require information from authors about some types of materials, experimental systems and methods used in many studies. Here, indicate whether each material, system or method listed is relevant to your study. If you are not sure if a list item applies to your research, read the appropriate section before selecting a response.

Materials & experimental systems

n/a	Involved in the study
☒	☐ Antibodies
☒	☐ Eukaryotic cell lines
☒	☐ Palaeontology and archaeology
☒	☐ Animals and other organisms
☒	☐ Clinical data
☒	☐ Dual use research of concern
☒	☐ Plants

Methods

n/a	Involved in the study
☒	☐ ChIP-seq
☒	☐ Flow cytometry
☒	☐ MRI-based neuroimaging

Plants

Seed stocks	n/a
Novel plant genotypes	n/a
Authentication	n/a
